# A novel protection liner to improve graft-tunnel interaction following anterior cruciate ligament reconstruction: a finite element analysis

**DOI:** 10.1186/s13018-020-01755-x

**Published:** 2020-06-23

**Authors:** Huizhi Wang, Min Zhang, Cheng-Kung Cheng

**Affiliations:** 1 Beijing Advanced Innovation Center for Biomedical Engineering, Beihang University, Beijing, 100083 China; 2 School of Biological Science and Medical Engineering, Beihang University, Beijing, 100083 China; 3 School of Biomedical Engineering, Shanghai Jiao Tong University, Shanghai, 200240 China

**Keywords:** ACL reconstruction, Graft wear, Bone tunnel enlargement, Implant, Finite element analysis, Biomechanics, Sports medicine

## Abstract

**Background:**

Deteriorated bone-graft interaction at the tunnel entrance following ACL reconstruction (ACLR) is considered one of the primary causes of long-term tunnel enlargement and graft wear. Methods have been introduced to improve the long-term outcome, such as novel graft materials or alternative fixation methods, but have been met with varying degrees of success. This study aims to design a protection liner to improve the bone-graft interaction at the tunnel entrances.

**Methods:**

A finite element model of a human cadaveric knee was used to simulate traditional ACLR and ACLR using the protection liner. Stress distribution around the tunnel entrances and on the ACL graft were calculated under a combined loading of 103 N anterior tibial load, 7.5 Nm internal tibial moment, and 6.9 Nm valgus tibial moment at a joint flexion angle of 20°. Results were compared between the traditional ACLR and ACLR using a double liner (femoral and tibial) setup, as well as between the ACLR using a double liner setup and a single liner (femoral side) setup. Different materials (PEEK, Ti-6Al-4V, CoCrMo) for the liner were also evaluated.

**Results:**

The traditional ACLR resulted in concentrated stress on the graft where it contacted the tunnel entrance. Correspondingly, there were stress concentrations at the distal posterior zone of the femoral tunnel entrance and medial posterior zone of the tibial tunnel entrance, while the other zones suffered from a stress reduction. Use of the protection liner reduced the stress concentration around the tunnel entrances by up to 89% and increased the stress at the unloaded zones by up to 106%. Also, stress concentration on the graft was slightly decreased (15.4 vs 15.1 MPa) after using the liner. The single liner setup increased the stress concentration around the tibial tunnel entrance. Stiffer materials improved the stress distribution around tunnel entrances but had little effect on the stress on the graft.

**Conclusions:**

The novel protection liner can improve the stress distribution on the graft and at the tunnel entrances, which may be beneficial for improving the clinical outcome of ACLR.

## Background

As a key stabilizer in the knee joint, the anterior cruciate ligament (ACL) is frequently injured, particularly during high speed movements such as in competitive sport [1]. Due to the poor healing capacity of the ACL, severe injuries often require ACL reconstruction (ACLR) [2], whereby, the injured native ACL is replaced with a graft. However, long-term complications have been reported following ACL reconstruction, among which bone tunnel enlargement and graft rupture have been reported with incidences of up to 72% and 22%, respectively [3, 4].

**Fig. 1:**
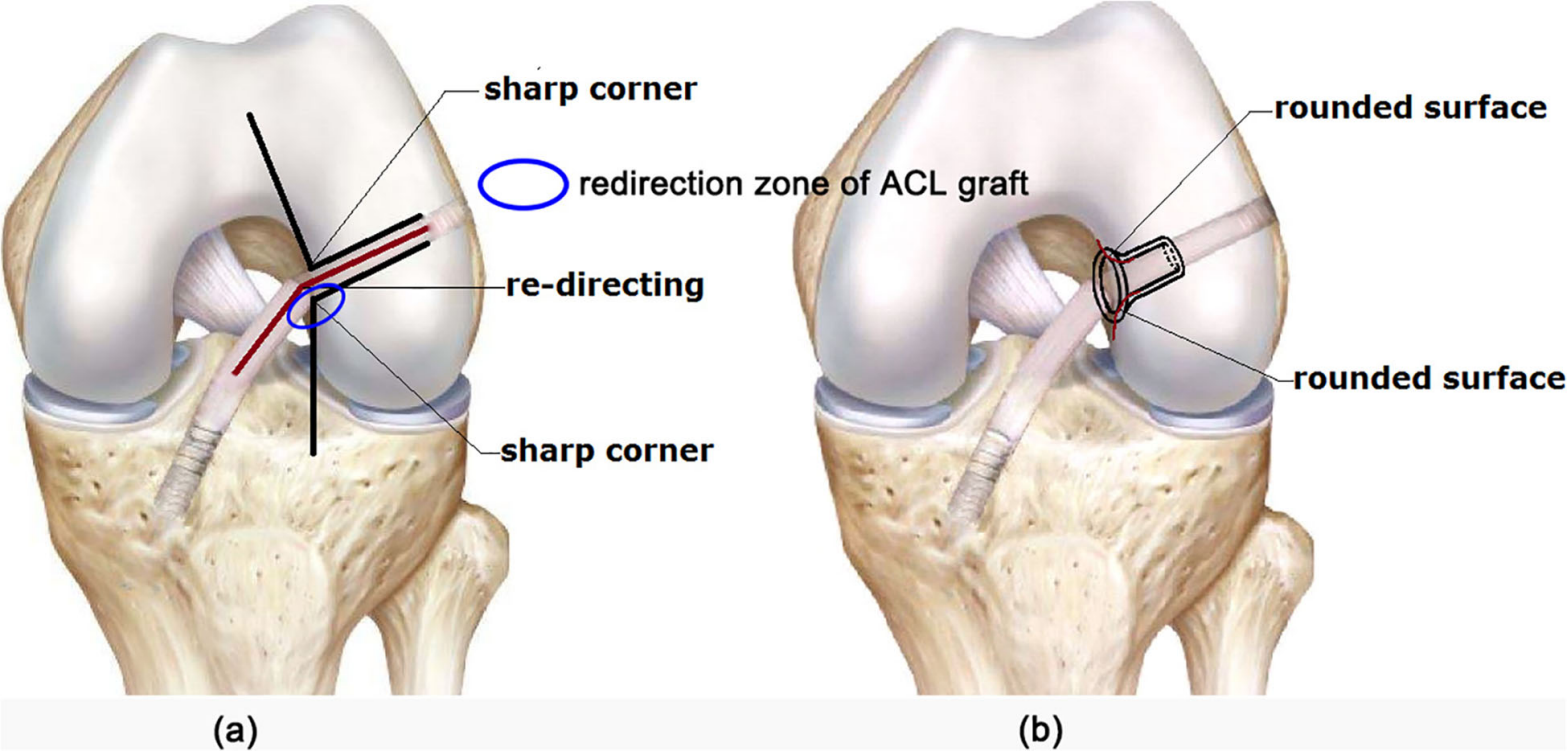
**a** Graft redirects at tunnel entrance in traditional ACL reconstruction. **b** Protection liner has a rounded surface which may improve the bone-graft interaction at tunnel entrance

Deteriorated interaction between the tunnel entrances and ACL graft is considered one of the primary causes of tunnel enlargement and graft wear/fatigue rupture. It has been shown [5] that at the tunnel entrance the graft redirects itself into the joint space (termed “redirection zone”) and comes into direct contact with the sharp rim of the tunnel (Fig. 1). This creates a stress concentration at this zone, while the other zones of the tunnel entrance may suffer from bone resorption and consequent tunnel enlargement because of a lack of stress. Also, high concentrated stress on the graft at the redirection zone may cause graft wear and long-term fatigue rupture.

Methods have been proposed previously for improving the interaction between the bone tunnel and graft. Pederzini et al. [6] stated that, by using a quadriceps tendon, which has a larger cross-sectional area than bone-patellar tendon-bone (BPTB) and semitendinosus/gracilis tendon (STG), the graft could be press-fit within the tunnels. This decreases the relative movement between the graft and bone tunnel and results in better stress distribution around the bone tunnel and on the graft. Similarly, Paessler et al. [7] used a press-fit fixation with hamstring (HS) grafts to refine the contact between the tunnel wall and the graft. During an ACLR revision surgery, Barrett and Brown [8] filled a femoral tunnel defect with a synthetic bone plug and stated that this would be beneficial for narrowing the enlarged tunnel and thus achieve a tighter contact against the graft.

However, few studies to date investigated methods for improving the interaction between the graft and sharp edges of the tunnel entrances. In this study, a protection liner was designed to cover the sharp rim of the tunnel entrances to improve the bone-graft interaction at this location. Finite element analysis was used to evaluate the biomechanical function of this design. The effects of using single (femoral side only) or double (femoral and tibial sides) liner setups were compared, and different materials for the liner were also evaluated. It was hypothesized that the protection liner could reduce stress concentrations at the tunnel entrances and on the graft and could transfer load to the previously unloaded zones of the tunnel entrances. It was also hypothesized that the double liner setup would function better than the single liner setup, and stiffer materials would provide better biomechanical functionality than softer materials. This study may serve to improve the clinical outcome of ACL reconstruction.

## Methods

### Design of the protection liner

**Fig. 2:**
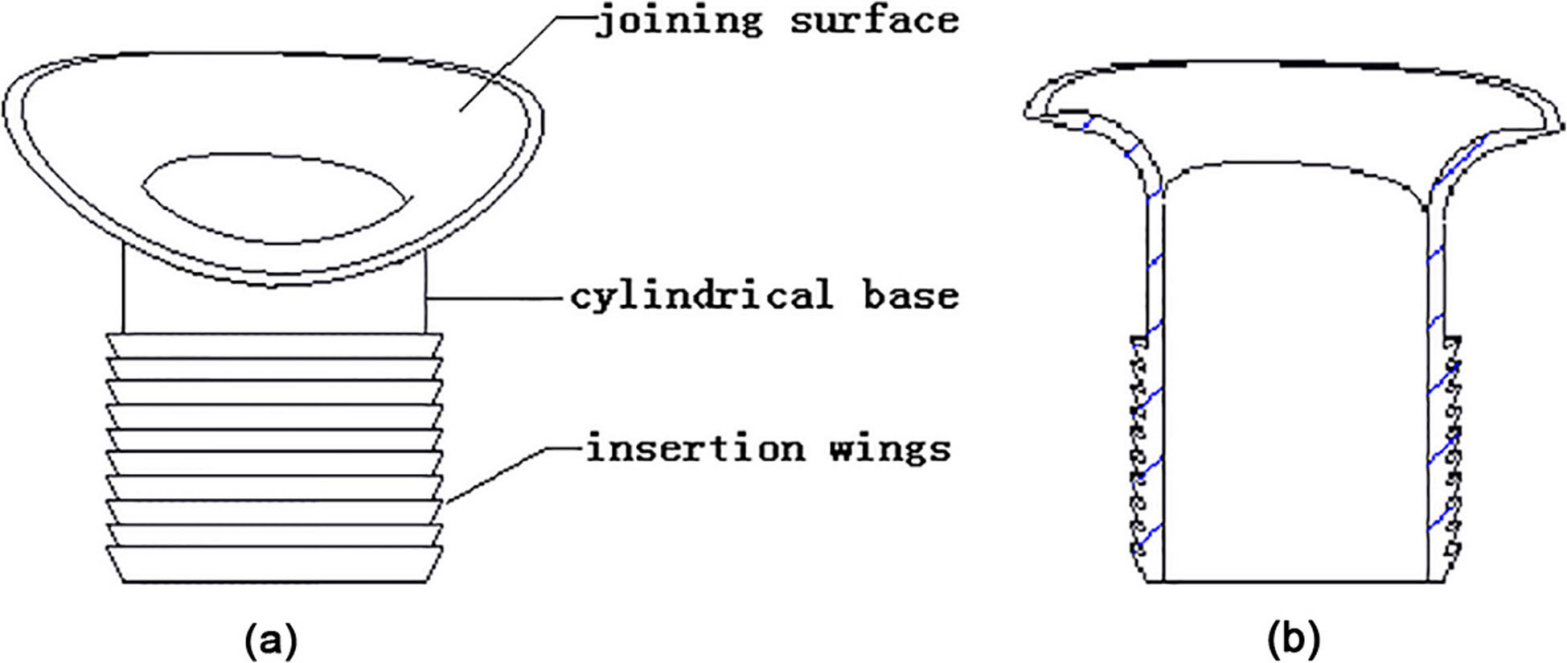
**a** Geometry of the protection liner. **b** Cross section of the protection liner

Figure 2 illustrates the design features of the protection liner, with a trumpet-shaped surface, a cylindrical base and a set of insertion wings (Fig. 2 a, b). During ACLR, the sharp bone tunnel entrances would first be rounded using a bone drill, then the protection liner would be inserted into the bone tunnels. The cylindrical base would be completely inserted into the bone tunnel and the trumpet-shaped surface would cover the tunnel entrance.

### Development and validation of a finite element model of human cadaveric knee

**Fig. 3:**
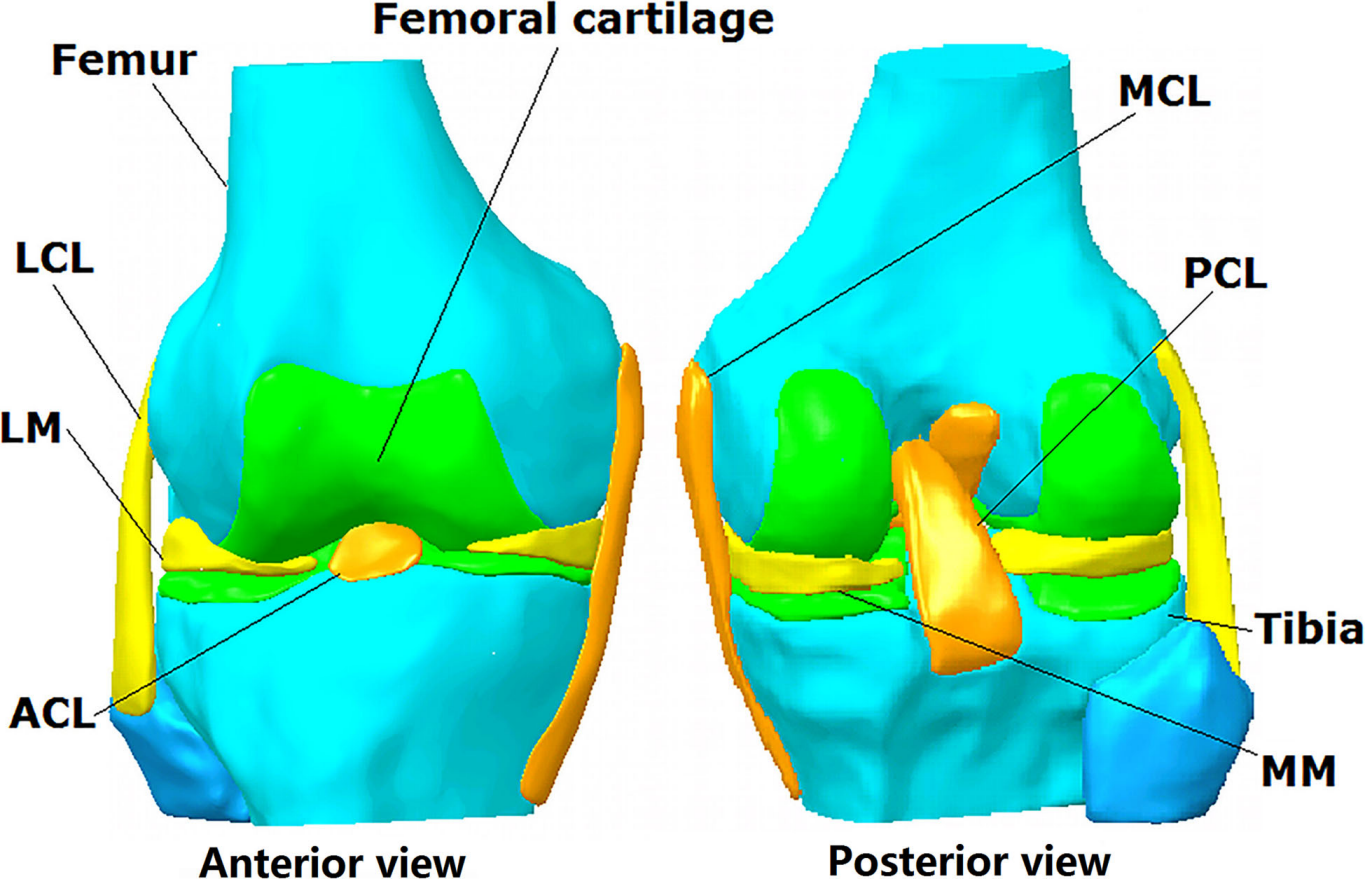
Reconstructed geometry of a human cadaveric knee

The geometry of a cadaveric knee (male, 45 years, 70 kg) was reconstructed from MRI images using Mimics 10.01 (Materialise N.V., Leuven, Belgium) (Fig. 3), with approval from the Committee for Oversight of Research and Clinical Training Involving Decedents. MRI scanning was performed using a FLASH pulse sequence, field of view measuring 12 cm  × 8 cm  × 10 cm, slice thickness of 0.2 mm, resolution of 0.2 × 0.2 × 0.2 mm/voxel, field strength of 3.0 T, and with TE = 26.3 ms and TR = 53 ms. The knee model consisted of the femur, tibia, cartilage, menisci (medial (M) and lateral (L)), ACL, posterior cruciate ligament (PCL), medial collateral ligament (MCL), and lateral collateral ligament (LCL) (Fig. 3).

A frictionless sliding contact was applied between the femoral and tibial cartilages, as well as between the femoral cartilage and the menisci. A tie contact was applied between ligaments and their bone insertions and between the cartilages and the corresponding bone surface. The mechanical properties of the joint tissues were defined according to literature [9, 10], as shown in Table 1. A pre-strain of 4% was defined for the ACL [10]. The model was meshed in HyperMesh 12.0 (Altair Engineering, Tokyo, Japan) using 4-node tetrahedron elements. A mesh convergence test was used to optimize the element size, whereby a 2.5-mm translational load was applied to the tibia while the femur was fixed at full extension of the joint in order to calculate the in situ force in the ACL. The element size was decreased until there was a negligible change in the in situ force in the ACL. The resulting element size was 1 mm. The model had a total of 659,251 elements.

**Table 1:** Definition of mechanical properties of tissues in the knee model

Tissue	Material type	Mechanical parameters
Bone	Isotropic elastic	Young’s modulus = 0.4 GPa, Poisson’s ratio *v* = 0.33
Cartilage	Isotropic elastic	Young’s Modulus = 5 MPa, Poisson’s ratio *v* = 0.46
Menisci	Orthotropic elastic	*E*_θ_ = 125 MPa, *E*_R_ = *E*_Z_ = 27.5 MPa, *G*_θR_ = *G*_θZ_ = 2 MPa, *G*_RZ_ = 10.34, *V*_θR_ = *V*_θZ_ = 0.1, *V*_RZ_ = 0.33
ACL	Isotropic hyperelastic	Veronda-Westmann: α = 0.3 MPa, β = 12.20
PCL	Isotropic hyperelastic	Veronda-Westmann: α = 0.18 MPa, β = 17.35
MCL and LCL	Isotropic hyperelastic	Mooney-Rivlin: C1 = 30.1 MPa, C2 = − 27.1 MPa

The model was then validated to be accurate in predicting joint kinematics and ACL forces under the following loadings at a joint flexion angle of 30°: (i) 134 N anterior tibial load; (ii) 10 Nm valgus tibial moment; (iii) 10 Nm internal tibial moment. The experimental data for model validation was obtained from cadaveric testing using a robotic/UFS system [11]. For the cadaveric testing, skin and soft tissues from the cadaveric knee were removed 10 cm proximal and distal to the joint line before testing, and then the femoral and tibial shafts were clamped to the robotic system. The femoral shaft was securely fixed while loading was applied to the tibial shaft. A passive path was first found by minimizing forces and moments in the joint from full extension to 90° of flexion. Joint positions throughout the passive path were considered as the reference positions for load application. After loading the knee, joint kinematics were recorded by the UFS testing system at each joint flexion angle. The ACL was then cut through its midsubstance, and the recorded joint kinematics were repeated. The forces experienced by the joint were recorded by the robotic/UFS testing system. In situ forces in the ACL were obtained using the principle of superposition. Experimental data under the loading conditions (i) and (ii) were obtained using the same sample for model building, while the data under the loading condition (iii) was obtained from a previous study [12]. Loading conditions (i), (ii), and (iii) were then applied to the finite element model and the resulting anterior tibial translation, internal tibial rotation, valgus tibial rotation, and in situ force in the ACL were calculated and compared with the experimental data.

### Evaluation of biomechanical function of the protection liner

To simulate traditional ACLR [13], the general procedure was to create the femoral and tibial tunnels and then fix ACL graft within the tunnels. Isometric points on the joint surface were used as intra-articular entry points for the bone tunnels. The femoral isometric point was defined as the center point of a circle prescribed on the posterior lateral femoral condyle with an arc of 140°, and the tibial isometric point was located half-way up the tibial plateau in the A-P direction and between the medial and lateral tibial ridges. The angles between the femoral tunnel axis and the sagittal and coronal planes were 45° and 25°, respectively, and the angles between the tibial tunnel axis and the transverse and sagittal planes were 55° and 30°, respectively. The ACL graft was modeled as cylindrical to simulate a LARS graft (LARS Company, Arc sur Tille, France), which is the most popular artificial ACL graft on the market [14]. A LARS 120-fiber graft was simulated and the corresponding diameter of the bone tunnel was 7.5 mm. A cylinder of diameter 7.5 mm and length 25 mm was used to simulate a titanium endoscrew, which was tied within the bone tunnel and tied to the ends of the ACL graft [15]. The stiffness of the graft was 323 N/mm and the titanium endoscrew had a Young’s modulus of 110 GPa and Poisson’s ratio of 0.35 [13, 16].

**Fig. 4:**
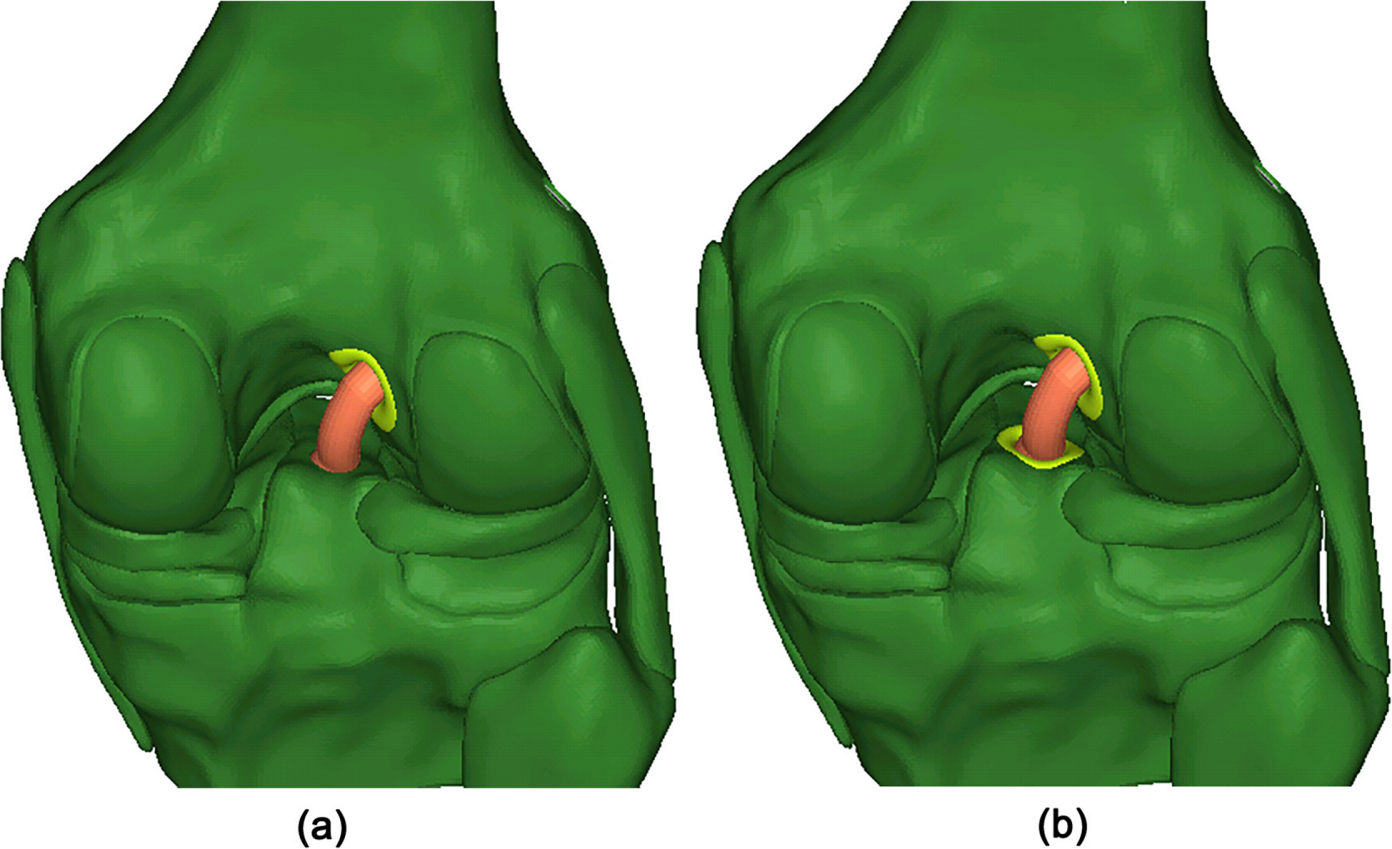
Simulation of ACLR using the novel protection liner. **a** ACLR using a single liner setup. **b** ACLR using a double liner setup

To simulate ACLR using a double liner setup, the protection liners were inserted into bone tunnels in the femur and tibia (Fig. 4). The outside diameter of the trumpet-shaped surface was 15 mm, the thickness of the cylindrical base was 0.5 mm, and the height of the insertion wings was 8 mm. To simplify the model, the geometry of the insertion wings was not simulated. The outside wall of the cylindrical base up to a height of 8 mm was tied to the tunnel wall to simulate a secure fixation of the protection liner. Frictionless sliding was defined between the graft and the protection liner, as well as between the graft and the tunnel wall. The material properties of the protection liner were defined as Ti-6Al-4V (Young’s modulus = 110 GPa and Poisson’s ratio = 0.35 [16]).

To evaluate the biomechanical function of the protection liner, the maximum anterior tibial load (103 N, 15% body weight), internal tibial moment (7.5 Nm, 1.1% body weight), and valgus tibial moment (6.9 Nm, 1% body weight) during normal gait [17] were applied to the intact model, traditional ACLR model, and the ACLR model using a double liner setup at a joint flexion angle of 20°. These loadings were chosen because they represent a worst case for tunnel-graft interaction during walking and put the majority of the strain on the ACL [18]. Stresses (von Mises stress, MPa) on the graft and at the tunnel entrances were calculated and compared among the different models. The von Mises stress is widely used to evaluate localized loading in tissues [19].

**Fig. 5:**
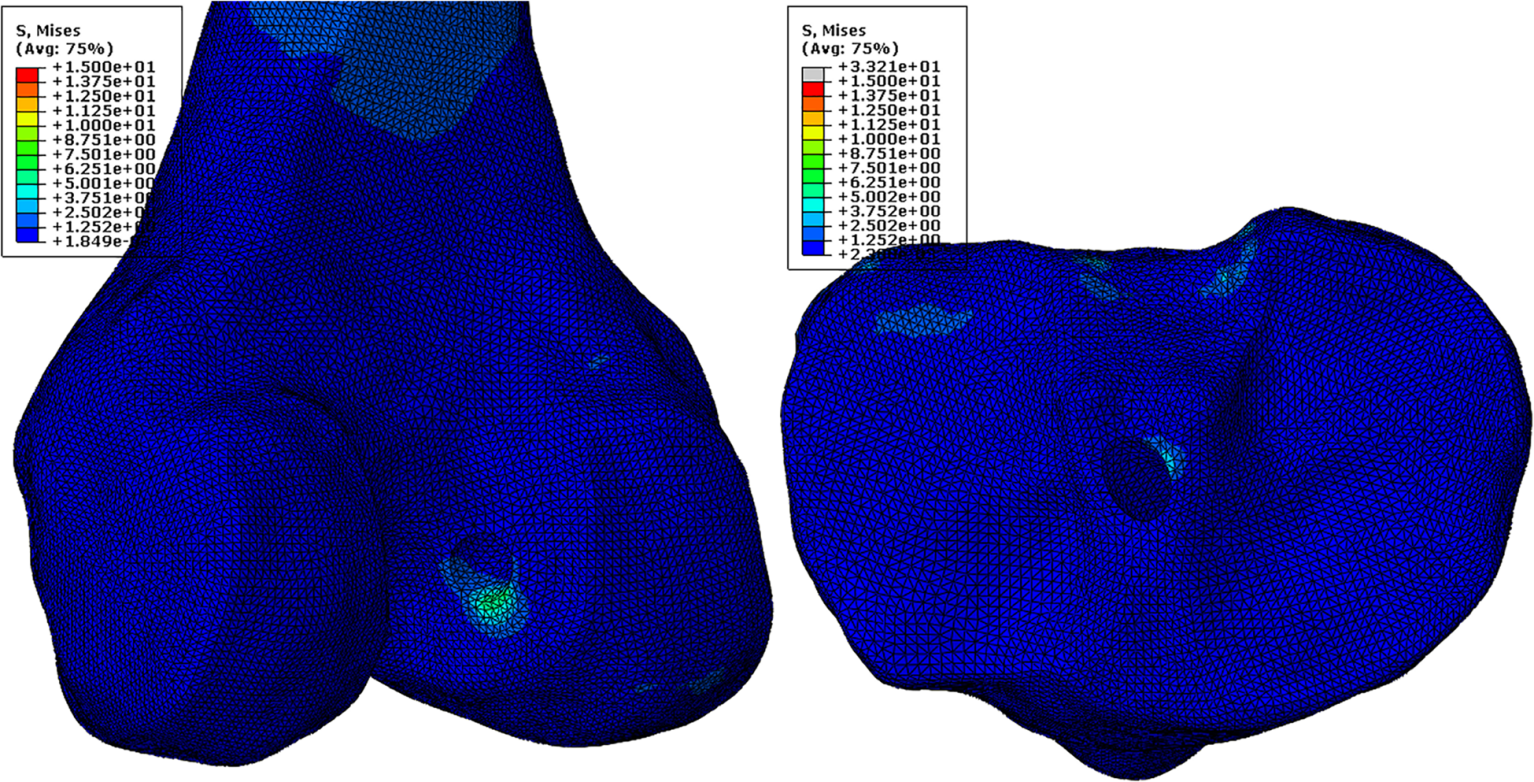
Femoral and tibial tunnel entrances divided into four zones

To describe the stress distribution around the tunnel entrances, the femoral and tibial tunnel entrances were divided into four zones within a circle of diameter 15 mm: anterior (A), posterior (Pos), proximal (Pro) and distal (D) zones for the femoral tunnel entrance, and anterior (A), posterior (P), medial (M), and lateral (L) zones for the tibial tunnel entrance (Fig. 5). For the femoral side, gray line 1 was first determined to pass through the femoral tunnel axis and ran parallel to the anatomical axis of the femoral shaft. Gray line 2 passed through the femoral tunnel axis and was perpendicular to gray line 1. Two perpendicular white lines were then plotted lying at 45° to the gray lines. For the tibial side, gray line 1 passed through the tibial tunnel axis and ran parallel to the femoral epicondylar axis [20].

### Single liner vs double liner

It has been reported that tunnel enlargement on the femoral side is typically more severe than on the tibial side [21]. Thus, this study compared the effectiveness of using a single (femoral side only) setup and double liner setup for ACLR (Fig. 4). Under the combined loading condition of 103 N anterior tibial load, 7.5 Nm internal tibial moment, and 6.9 Nm valgus tibial moment, the maximum von Mises stress on the graft and at the tunnel entrances were calculated and compared between the two groups.

### Evaluation of liner material

Three materials commonly used in medical devices were used to construct the liner: PEEK (Young’s modulus = 3500 MPa), Ti-6Al-4V (Young’s modulus = 110 GPa), and CoCrMo (Young’s modulus = 240 GPa). The loading condition of 103 N anterior tibial load combined with 7.5 Nm internal tibial moment and 6.9 Nm valgus tibial moment was applied to the double liner ACLR models with different liner materials at a joint flexion angle of 20°. The maximum von Mises stress at the tunnel entrances and on the graft were calculated and compared among the three groups.

## Results

### Validation of the finite element model

Compared with the data from robotic testing, the finite element model had differences of 0.1 mm, 1°, and 1 N for anterior tibial translation, valgus tibial rotation, and in situ force in the ACL, respectively (Table 2). The results for valgus tibial rotation and in situ force in the ACL were within the range of experimental data reported in literature [12].

**Table 2:** Anterior tibial translation, valgus tibial rotation, internal tibial rotation, and in situ force in the ACL obtained from robotic testing and finite element model under the loading condition (i) 134 N anterior tibial load; (ii) 10 Nm valgus tibial moment; (iii) 10 Nm internal tibial moment at a joint flexion angle of 30°

	134 N Anterior tibial load		10 Nm valgus tibial moment		10 Nm internal tibial moment	
	Anterior tibial translation (mm)	In situ force in the ACL (N)	Valgus tibial rotation (°)	In situ force in the ACL (N)	Internal tibial rotation (°)	In situ force in the ACL (N)
Experimental (robotic)	5.1	124	5	42	22 ± 3	41 ± 21
Computational (finite element)	5.2	123	4	41	19	62

### Evaluation of biomechanical function of the protection liner

**Fig. 6:**
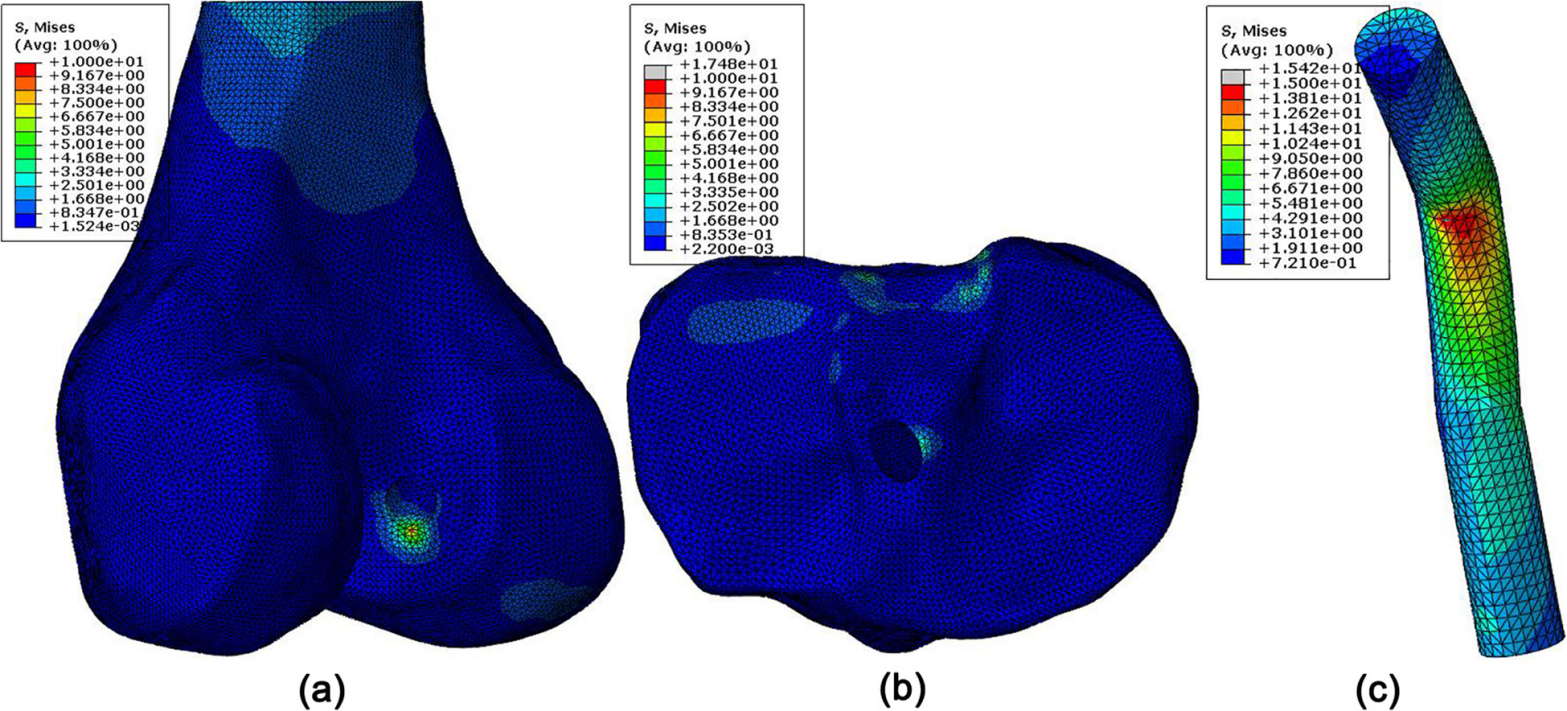
Stress distribution around tunnel entrances and on the ACL graft following traditional ACLR

Stress concentrations were found at the distal zone of the femoral tunnel entrance in the traditional ACLR model, while the other zones of the tunnel entrance showed lower stress levels (Fig. 6a). On the tibial side, there was a stress concentration at the posterior medial section of the tunnel entrance (Fig. 6a). There was also an obvious stress concentration on the ACL graft close to the femoral tunnel entrance (Fig. 6c).

Table 3 shows the maximum von Mises stresses at each zone of the tunnel entrances in the traditional ACLR model, ACLR using the protection liner and the intact model. Following traditional ACLR, the maximum stress at the distal and posterior zones of the femoral tunnel entrance increased by 2–7 times over the intact joint, while the anterior and proximal zones saw a reduction in stress of 74–83%. Similarly, the maximum stress at the medial and posterior zones of the tibial tunnel entrance increased 2–3 times over the intact joint, while that at the anterior and lateral zones decreased by 21–74%. The maximum stress on the ACL graft at its redirection zone was 15.4 MPa. After inserting the protection liner, stresses at the anterior and proximal zones of the femoral tunnel entrance increased in comparison to the traditional ACLR (0.94 vs 1.29 MPa and 0.35 vs 0.70 MPa), bringing the values closer to those from the intact joint (5.56 and 1.33 MPa). Similarly, stresses at the anterior and lateral zones of the tibial tunnel entrance increased after inserting the protection liner (0.29 vs 0.45 MPa and 0.38 vs 0.46 MPa). Meanwhile, the stress decreased at the distal and posterior zones of the femoral tunnel entrance (4.54 vs 0.52 MPa and 0.64 vs 0.26 MPa) and at the medial zone of the tibial tunnel entrance (1.75 vs 0.33 MPa). However, stress concentrations at the posterior zone of the tibial tunnel entrance were not alleviated (0.66 vs 0.78 MPa). Also, use of a protection liner caused a slight decrease in contact stress on the ACL graft in comparison to traditional ACLR (15.4 vs 15.1 MPa).

**Table 3:** Maximum von Mises stress (MPa) at different zones of the tunnel entrances in each model under the loading condition 103 N anterior tibial load + 7.5 Nm internal tibial moment + 6.9 Nm valgus tibial moment at a joint flexion angle of 20°

	Femoral tunnel entrance				Tibial tunnel entrance			
	A zone	D zone	Pos zone	Pro zone	A zone	L zone	M zone	P zone
Intact	5.56	0.56	0.21	1.33	1.13	0.48	0.47	0.25
Traditional ACLR	0.94	4.54	0.64	0.35	0.29	0.38	1.75	0.66
Double liner (Ti-6Al-4V, 110 GPa) aided ACLR	1.29	0.52	0.26	0.70	0.45	0.46	0.33	0.78
Double liner (PEEK, 3500 MPa) aided ACLR	0.83	1.67	0.47	0.41	0.25	0.22	0.71	0.46
Double liner (CoCrMo, 240 GPa) aided ACLR	1.32	0.64	0.26	0.74	0.48	0.50	0.32	0.79
Single liner aided ACLR	1.37	0.73	0.29	0.72	0.27	0.37	1.52	0.54

### Single liner vs double liner

Referring to Table 3, the use of a single protection liner resulted in similar stress at the femoral tunnel entrance as the double liner setup (1.29 vs 1.37 MPa, 0.70 vs 0.72 MPa, 0.52 vs 0.73 MPa, and 0.26 vs 0.29 MPa, for the anterior, proximal, distal, and posterior zones, respectively). For the tibial side, the single protection liner produced lower stress at the anterior, lateral, and posterior zones of the tibial tunnel entrance (0.45 vs 0.27 MPa, 0.46 vs 0.37 MPa, and 0.78 vs 0.54 MPa) but greater stress at the medial zone (0.33 vs 1.52 MPa). The single protection liner resulted in similar contact stress on the graft with the double protection liner (15.1 vs 14.9 MPa).

### Effect of liner material on its function

As shown in Table 3, as the stiffness of the protection liner increased, the maximum stress at the anterior and proximal zones of the femoral tunnel entrance increased, as well as that at the anterior and lateral zones of the tibial tunnel entrance. The liners composed of Ti-6Al-4V (110 GPa) and CoCrMo (240 GPa) resulted in lower stress than the PEEK (3500 MPa) liner at the distal and posterior zones of the femoral tunnel entrance, and at the medial zone of the tibial tunnel entrance. The maximum contact stress at the redirection zone of the ACL graft was the same for the different liner materials (15.1 MPa).

## Discussion

This study evaluated whether inserting a protection liner at the entrance to the bone tunnels could improve the interaction between the bone tunnel entrances and ACL graft following ACLR.

The results showed obvious stress concentrations in the region where the ACL graft contacts the tunnel entrances, particularly on the femoral side. Stress concentrations on the graft at its redirection zone indicated a heightened risk of graft wear [22, 23]. The other non-contacted zones of the tunnel entrances showed a reduction in stress in comparison to the intact (non-ACLR) model, which was in agreement with results reported by Jagodzinski et al. [22]. Stress at the femoral tunnel entrance was highest at the distal zone, followed by the anterior zone, which corresponds with the previous research [5]. Compared with the intact joint, there was an obvious reduction in stress at the anterior and proximal zones of the femoral tunnel entrance, as well as at the anterior and lateral zones of the tibial tunnel entrance following traditional ACLR, which may cause bone resorption and subsequent tunnel enlargement at those regions. Tibial tunnel enlargement has been reported to be larger in the sagittal plane than the coronal plane [24], which was in agreement with the current study whereby the greatest reduction in stress occurred at the anterior zone of the tunnel entrance. Similarly, the current results showed that the largest stress reduction at the femoral tunnel entrance happened at its anterior zone, which may explain the frequent reports of an anterior shift in the femoral tunnel [25].

Compared with traditional ACLR, inserting the protection liner increased the stress at the anterior and proximal zones of the femoral tunnel entrance, and at the anterior and lateral zones of the tibial tunnel entrance, while caused alleviated stress concentration at the other zones of the tunnel entrances, suggesting a more evenly distributed stress on the bone, which may decrease the risk of bone resorption and consequent tunnel enlargement. The protection liner also reduced the stress on the graft slightly (15.4 vs 15.1 MPa), likely lowering the risk of graft wear.

The single liner setup (femoral side only) showed lower stresses at the anterior and lateral zones of the tibial tunnel entrance than both the double liner setup (femoral and tibial side) and the traditional ACLR model. Lowering the stress in these regions below normal physiological levels may reasonably be expected to induce bone resorption, and thus it is not recommended to use the single liner setup demonstrated in this study. The maximum stress on the graft was similar between the single and double liner setups (15.1 MPa vs 14.9 MPa).

Increasing the stiffness of the protection liner resulted in a more evenly distributed stress around the tunnel entrances and had no effect on the maximum stress on the graft. Compared with the PEEK (3500 MPa) liner, the Ti-6Al-4V (110 GPa) liner caused an increase in stress at the anterior and proximal zones of the femoral tunnel entrance, as well as that at the anterior and lateral zones of the tibial tunnel entrance by up to 1.15 MPa, while the CoCrMo (240 GPa) liner increased the corresponding stresses by a further 0.12 MPa over the Ti-6Al-4V liner. This suggests that once a certain threshold has been reached, further increasing the stiffness of the liner does not noticeably improve its biomechanical function. However, both Ti-6Al-4V and CoCrMo offer superior results over the PEEK liner for improving the stress distribution around the tunnel entrances.

There are some limitations to this study that should be noted: (i) The loading condition used in this study was static and cannot represent the actual loading conditions during gait, but was used here to evaluate the basic function of the protection liner. (ii) The geometry of the wings was not included in the model, but the ideal function of it was represented with a tie interaction between the cylindrical base and the tunnel wall. Future studies using animal or human cadaveric specimens should test the practical function of the wings for liner fixation. (iii) Another limitation is that friction was not defined between the graft and the bone tunnel, which might have an effect on the results. According to Wan and Hao [26], the graft-tunnel friction had little effect on the distribution and maximum value of principle strain on the graft, but decreased the maximum strain for the bone tunnel from 0.015 to 0.011 with the increasing of friction coefficient (from 0 to 0.3), although the strain distribution at bone tunnel aperture was little affected. (iv) Since biological integration between bone tunnel and the ACL graft has been reported to be poor [27], it was considered acceptable in this study that the liner acted to separate the graft from surrounding bone. The protection liner might also shield the tunnel aperture from synovial fluid infiltration, thus reducing the severity of the Windshield Wiper Effect often seen with traditional ACLR. However, future studies may consider making the liner porous to facilitate cell transportation and allow a level of graft-bone integration. (v) Despite the common occurrence of tunnel enlargement following ACLR and research devoted to this subject, the process leading to enlargement is thought to be multifactorial and complex. This study evaluated the problem from a biomechanical perspective and attempted to reduce bone resorption caused by lack of stress. While biological factors may also lead to tunnel enlargement, these factors were not evaluated in this study. Future studies may look at evaluating other potential inputs to tunnel enlargement (e.g., biological sources) and attempt to reduce these in a combined effort.

## Conclusions

In conclusion, the protection liner designed in this study can improve the interaction between the graft and bone tunnel entrances after ACLR. The use of a double liner setup is suggested over a single liner setup and stiffer materials like Ti-6Al-4V and CoCrMo are superior to softer materials like PEEK for improving the function of the protection liner. This study may serve to improve the clinical outcome of ACLR.

## Data Availability

The data used in the current study are available from the corresponding author upon reasonable request.

## Abbreviations

ACL: Anterior cruciate ligament
ACLR: Anterior cruciate ligament reconstruction
M: Medial
L: Lateral
A: Anterior
P: Posterior
Pro: Proximal
Pos: Posterior
D: Distal
MCL: Medial collateral ligament
LCL: Lateral collateral ligament
PCL: Posterior cruciate ligament
